# Short tandem repeat near hypoxia response element (HRE) instead of HRE genetic variants in promoter calcitonin receptor-like receptor (CRLR) gene as risk factor in severe preeclampsia: a preliminary study

**DOI:** 10.1186/s13104-020-05437-z

**Published:** 2021-01-07

**Authors:** Herlambang, Amelia Dwi Fitri, Ahmad Syauqy, Yuwono, Anggelia Puspasari, Rina Nofri Enis, Ahmad Faried

**Affiliations:** 1 Department of Obstetrics and Gynaecology, Division of Fetomaternal, Faculty of Medicine and Health Sciences, University of Jambi (FKIK UNJA)-Raden Mattaher General Hospital (RSRM), Jl. Letjen Soperapto 33, Jambi, 36122 Indonesia; 2 FKIK UNJA-RSRM, Jambi, Indonesia; 3 Department of Medical Biology and Biochemistry FKIK, UNJA-RSRM, Jambi, Indonesia; 4 Department of Microbiology, Faculty of Medicine, University of Sriwijaya, Palembang, Indonesia; 5 Department of Anatomy, FKIK UNJA-RSRM, Jambi, Indonesia; 6 Oncology and Stem Cell Working Group, Faculty of Medicine, Universitas Padjadjaran-Dr, Hasan Sadikin Hospital, Bandung, Indonesia

**Keywords:** Preeclampsia, CRLR, CA repeat, HRE, CACA box polymorph, Gene promoter

## Abstract

**Objective:**

Calcitonin receptor-like receptor (CRLR) regulates vasoconstriction and dilatation; the expression increases during hypoxia via activation of hypoxia response element (HRE) in CRLR gene promoter region. Variant in HRE, as well short tandem repeat (STR) variants near HRE in CRLR alters the gene expression. This study focused on a case–control study to investigate the expression of genetic typing CLRL promoter variant in pregnant women with severe preeclampsia and normal pregnancies, we also tried to describe interesting findings of the genetic expression in anemic patients in the severe preeclampsia group. Our aimed to observe the correlation of CRLR gene promoter variant and anemia in severe preeclampsia.

**Results:**

There was no nucleotide variant in HRE; CACA box prior to HRE varied in length (15–24); CACA box with length > 20 was used as cut off point. Hb was lower in CACA box length ≥ 21 (10.33 ± 1.57) vs. < 21 (11.01 ± 1.67; *p* = 0.391). CACA box polymorphism and anemia were correlated in severe preeclampsia (*p* = 0.005) OR 0.038 (CI 0.003–0.544); not in normal (*p* = 0.069).

## Introduction

Hypertension and anemia are common disorders in pregnancy. Preeclampsia is one of hypertensive disorders and anemia in preeclampsia patient can be related to HELLP syndrome or isolated pathological condition, in case of simultaneous occurrence, the risk of maternal mortality and morbidity are higher. Preeclampsia and anemia affect mother and baby outcomes due to chronic hypoxia [1–4]. Adrenomedullin signaling plays important role in vessel dilatation and constriction in pregnancy; depends on the interaction of calcitonin gene related peptide (CGRP), calcitonin receptor-like receptor (CRLR) and receptor activity modifying protein (RAMP). CRLR expression in preeclampsia reported to be altered and increased in hypoxia condition including hypertension and anemia, although in preeclampsia some studies said otherwise [5–9]. CRLR encoded by *CRLR*, located in 2q32.1. CACA box length, a STR in *CRLR* and *HRE* structure close to transcription initiation site, play role in protein expression as in other gene and affected phenotype. In other gene, CACA box length, HRE genetic variant alter protein expression and related to many pathologic condition. CACA box with (AC) 17–39 repeat in the promoter region of the HO-1 gene, polymorphisms of which is associated with cardiovascular diseases, Parkinson’s, cancer and preeclampsia [10, 11]. HRE nucleotide variants in several genes affected gene expression via hypoxia induced factor (HIF) binding [12, 13]. In preeclampsia, chronic hypoxia can occur. Anemia in pregnancy can also lead to chronic hypoxia. Anemia and Preeclampsia affected mothers and baby outcomes, when it happened simultaneously related to higher maternal mortality and morbidity. There have been several studies conducted to investigate CACA box length and HRE genetic variants in CRLR gene promoter in patients with severe preeclampsia, but studies on the review of genetic expression of severe preeclampsia by looking at hemoglobin levels in severe preeclampsia patients have never been done [1–4].

## Main text

### Material and methods

#### Study subject and sampling collecting

This study obtained permission from the Faculty of Medicine and Health Sciences, Jambi University ethical research committee. We conducted case–control study involving 40 patients, separated into two groups. Case group (20 patients) with severe preeclampsia and control group (20 patients) with normal pregnancy. The study participants gave birth in Raden Mattaher Hospital, Jambi, Indonesia. The inclusion criteria for severe preeclampsia based on systolic blood pressure ≥ 160 mm Hg or diastolic blood pressure ≥ 110 mm Hg with or without proteinuria after 20 weeks of gestation. Hemoglobin level, demographic data, and obstetric history were ascertained based on hospital records and reconfirmed to the patients when placental tissue and maternal blood samples were taken. The exclusion criteria are multiple pregnancies, premature labor, previous history of chronic diseases and acute inflammatory diseases. Peripheral venous blood collected from mother before gave birth in an etilenadiaminatetraasetat acid (EDTA)-coated tube for DNA and PCR. PCR and DNA sequencing used for genotyping placenta tissue and maternal blood in both group. We investigate hypoxia-induced factor (HIF) to hypoxia response element (HRE) in CRLR gene promoter region. Variant in HRE altered HIF, as well short tandem repeat (STR) variants near HRE in CRLR. CACA box is STR, genetic variants determined transcriptional complexes located outside the STRs related to difference in distance to transcription start site.

#### Isolation of DNA, PCR and genotyping

DNA was extraction from peripheral blood sample solid phase method by Chelex-100 resin Catalogue No 7610010, purity DNA extraction determined with Spectrophotometry. Polymerase Chain Reaction (PCR) used to amplified promoter CRLR genome segment suspected, forward primer 5′ GGAGGAACAGCACCCAATTA 3′and reverse primer 5′ GCTGGCTTTCACCTTGA CTG 3′, The PCR product was 304 base pairs, and run in electrophoresis. Denaturation temperature was 94℃ for 50 s, annealing temperature was 59℃ for 50 s for 35 cycle and extension in 70℃ for 10 s. Genotyping of PCR product used Sanger Sequencing method, the sequence data was analyzed by Bio-edit VII software by matching the data with reference sequence from Gene Bank. Gene ID 10203 was used as reference sequences.

#### Statistical analysis

Participant characteristic analyzed with t-test for quantitative- and chi square for qualitative-data. Cut off point CACA box length polymorphism based on mean value of participants. Correlation of CACA box length and preeclampsia analyzed with chi square. Subgroup analysis was perform to find conditions related to preeclampsia with CACA box genetic variant. A *p* < 0.05 was considered significant. Statistical analyses were performed with IBM SPSS statistic 23 software.

### Results

#### Baseline characteristic study participants

We assessed 40 pregnancy women who gave birth at Raden Mattaher Hospital, Jambi, Indonesia within 2017. All study participants were full term pregnancy at delivery, Malayan ethic resided at Jambi Province and had been signed informed consent. We modeled the baseline characteristic of study participants. In model of severe- vs. normal-pregnancy, age distribution was older, leucocytes count was lower, thrombocytes count slightly higher, anemia was lower in severe pregnancy group but statistically insignificant. In severe preeclampsia group we found higher systolic blood pressure, higher diastolic blood pressure, higher hemoglobin level, lower anemia frequency, higher pathologic proteinuria and lower baby birth weight, all of the variables were statistically significant (Table 1).

**Table 1:** Baseline characteristic study participants

Characteristic	Normal pregnancy (n = 20)	Severe preeclampsia (n = 20)	*p* value	Anemia (n = 21)	Normal Hemoglobin (n = 19)	*p* value
Age* (years)	27.05 ± 5.91	30.1 ± 5.84	0.109	26.38 ± 5.01	31.00 ± 6.19	*0.013*
Systolic blood pressure** (mmHg)	113.5 ± 9.33	168 ± 8.33	*0.000*	125.23 ± 24.62	157.89 ± 23.47	0.665
Diastolic blood pressure** (mmHg)	80 ± 7.25	103.5 ± 4.89	*0.000*	86.19 ± 10.71	97.89 ± 13.57	0.501
Hemoglobin** (gr/dl)	9.91 ± 1.74	11.53 ± 1.07	*0.001*	9.65 ± 1.49	11.9 ± 0.77	0.804
Leucocytes count* (cell/dl)	14.85 ± 3.98 × 10^3^	13.93 ± 4.28 × 10^3^	0.485	14.99 ± 4.42	13.72 ± 3.74	0.334
Thrombocytes count** (cell/dl)	299.15 ± 92.34 × 10^3^	299.4 ± 65.53 × 10^3^	0.862	308.28 ± 86.27	289.32 ± 86.27	0.099
Anemia (%)	75%	30%	*0.011*	–	–	–
Severe pre-eclampsia (%)	–	–	–	30% (n = 6)	75% (n = 15)	*0.011*
CACA box length ≥ 21	55% (n = 9)	30% (n = 5)	0.201	64.7% (n = 11)	43.5% (n = 10)	0.313
Pathologic proteinuria (%)	0%	80% (n = 16)	*0.000*	31.3% (n = 5)	66.7% (n = 16)	0.061
Baby weight* (gram)	3162.5	2527.5	*0.000*	2971.43	2705.26	0.062

^*^ Age, leucocytes count, baby weight have normal distribution, Independent t-test was used

^**^ SBP, DBP, Hb, TC have abnormal distribution after transform, Man–Whitney test was used

Grouping of patients based on hemoglobin level (cut off point for hemoglobin level was 11 mg/dl). In this grouping, systolic blood pressure was higher, diastolic blood pressure was higher, hemoglobin level was higher, leucocytes count was lower, thrombocytes count was lower, pathologic proteinuria was higher and baby weight was lower in normal hemoglobin level, all variables were not statistically significant. We also found that older age and higher severe preeclampsia frequency in normal hemoglobin level group, both statistic significant (Table 1).

#### Genotyping genetic variants

**Fig. 1:**
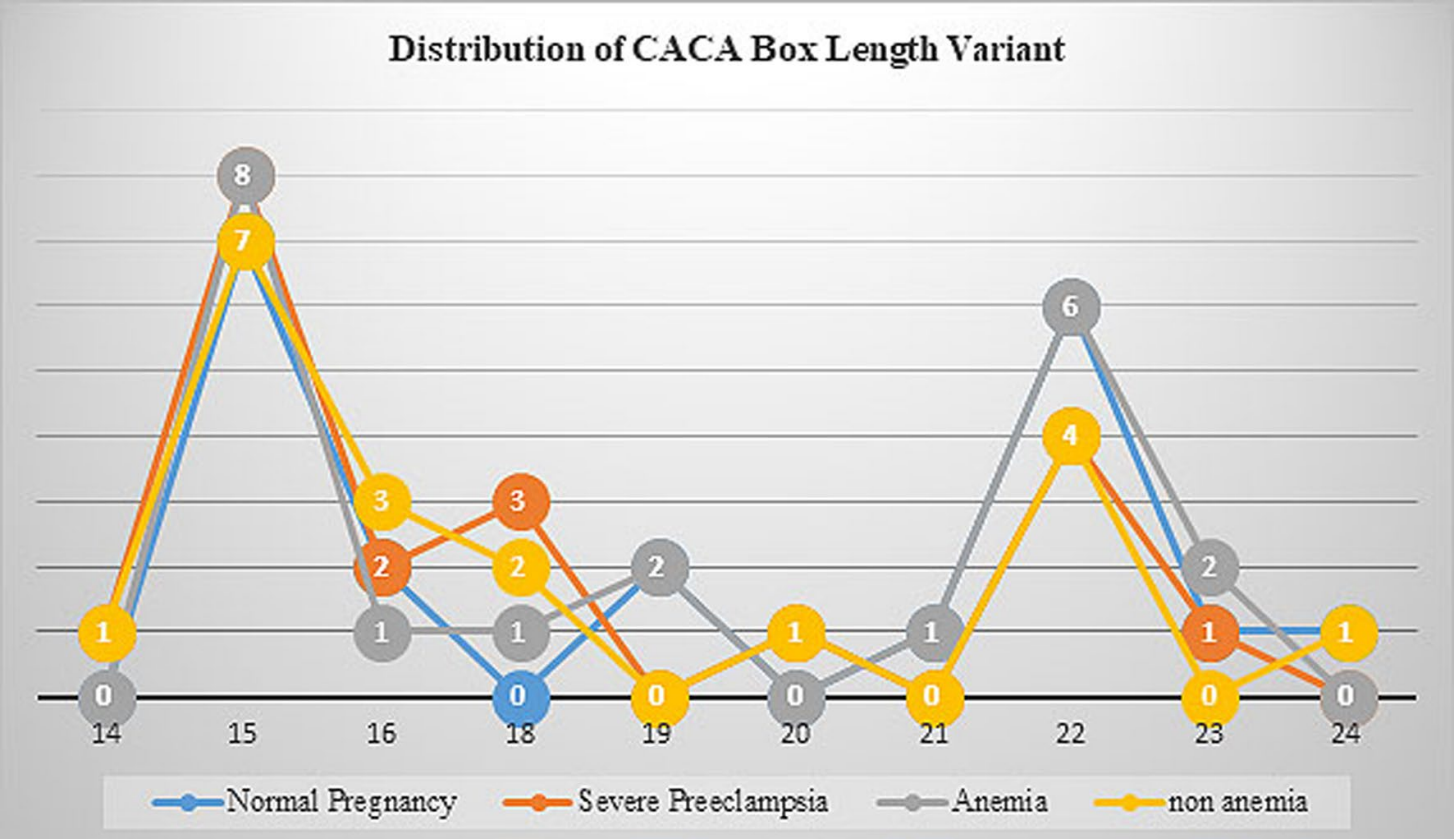
Distribution of CACA box among the study participants. Vertical axis showed the study participants frequency and vertical axis showed CACA box polymorphism, blue line for normal pregnancy, orange line for severe preeclampsia, grey line for anemia and yellow line for non-anemia

HRE in *CRLR* promoter is located downstream just after CACA box and in close proximity to transcriptional start site. HRE is gene promoter sequences (5′-CACGC-3′) where HIF bind and effected gene expression; no polymorphism of nucleotide sequences found in our population (Additional file 1). Alteration of transcription factor binding in previous study related to STR genetic variants and we found STR as CACA box genetic variant near HRE sequences. Based on genotyping in our study population we found that CACA box length variant of 14–24 CACA box length. The most frequent of CACA box length in our population was 15 and 22 (Fig. 1). The investigation of CACA box length variants in *CRLR* has never been done, we use 21 CACA box length as cut off point to classified polymorphism and normal. Normal variant defined as CACA box length was < 21 and polymorphism as CACA box length was ≥ 21. Polymorphism frequency was 42.5% in our population. In severe preeclampsia, polymorphism frequency was lower (30%) than in normal pregnancy (55%) although statistically not different (*p* = 0.201). At the further analysis in the severe preeclampsia group, we reveal an interesting finding, polymorphisms are higher in the anemia group (64.7%) compared to the non-anemia (43.5%) group with statistically significant (*p* = 0.005) with OR 0,038 (CI 0.003–0.544), showed in Table 2.

**Table 2:** Subgroup Analysis of Severe Preeclampsia group based on Hemoglobin level

Characteristic	Normal pregnancy			Severe preeclampsia		
	Poly	WT	*p* value	Poly	WT	*p* value
Hemoglobin						*0.014*
Anemia (%)	5 (25%)	10 (50%)	0.069	4 (20%)	2 (10%)	OR (0.038)
Normal (%)	4 (20%)	1 (5%)		1 (5%)	13 (65%)	CI 0.003–0.544
Hemoglobin (gr/dl)	10.17 ± 1.84	9.7 ± 1.71	0.556	10.84 ± 1.40	11.76 ± 0.86	0.096

Hemoglobin data showed abnormal distribution after transform, Man–Whitney test was used; *p* value < 0.05 as significant statistically

Grouping of patients based on CACA box length found that there was no statistically difference between all variables. Hemoglobin level was lower (10.41 ± 1.67) in polymorphic group than in normal variant (10.88 ± 1.63; *p* = 0.391). Anemia frequency was higher in polymorphic group (64.7%) than in normal variant (43.5%) and not statistically significant (*p* = 0.313). Subgroup analysis showed that *CRLR* polymorphism was a risk factor for anemia with OR 0,038 (CI 0.038–0.544) in severe preeclampsia, showed in Table 2. Addition of CACA box analysis, our study participant genotyping showed no nucleotide sequences variances. It is indicated that HIF binding in CRLR gene promoter influenced by genetic variants of CACA box length instead of HRE DNA sequences pattern.

### Discussion

*CRLR* codes CRLR protein, a GCPRs family. CRLR is receptor for CGRP ligand; activated by protein called RAMPs and recognized as integral component of the adrenomedullin signaling system that regulated vasodilatation, vessel permeability, inhibition endothelial cell apoptosis and promotion of angiogenesis. Adrenomedullin signaling play important role in preeclampsia pathophysiology [5, 7–9]. Preeclampsia, a hypertensive disorder spectrum caused by dysregulation of vasoconstriction and vasodilatation. Anemia is one of the hematologic problems in pregnancy because of the physiologic changes in pregnancy. Anemia in preeclampsia can be part of preeclampsia or a separated pathologic condition, both related to chronic hypoxia and increased maternal and perinatal mortality [1–3].

Genetic variant of *CRLR* alter its expression, CRLR alteration found in preeclampsia and anemia related to hypoxic condition. HRE in other gene regulated protein transcription through binding with HIF transcription factor. HRE gene variant associated to compensation mechanism in hypoxia as well in anemia [12, 13]. There was no HRE nucleotide mutation in our population. It might indicate that HIF binding in CRLR gene promoter influenced by genetic variants of CACA box length instead of HRE DNA sequence motives.

Regulation of transcription factor in hypoxia related to other DNA sequence motives like CACA box length upstream 5′ from TSS. CACA box is STR that consist AC repeat difference and predominant in some gene promoter such as CRLR. STR does not bind transcription factor but determine other nucleotide sequences to bind transcription factor. The difference of CACA box length relates to difference of distance to TSS. It is related to difference of transcription rate and might influence gene expression. Higher protein expression found in shorter CACA box length in some gene [10, 11]. We showed lower hemoglobin level in polymorphic group. In addition, when we stratified severe preeclampsia into two groups anemia and non-anemia, we found polymorphic more frequent in the anemia vs. non-anemia group. We suggest polymorphic group who had longer CACA box length and correlated with lower CRLR expression might compensated mechanism of hypoxia related to anemia and preeclampsia. They maintain vasoconstriction in condition of lower hemoglobin level, although the mechanism correlated with these two were not clear yet.

### Conclusion

There was no HRE genetic variants in our study population. Anemia in severe preeclampsia was more frequent in polymorphic group, who had longer CACA box length. Larger sample are needed to see correlation of genetic variant of CRLR gen promoter with severe preeclampsia and anemia.

## Limitation of the study

The limitation of this preliminary study was small sample size; further sample with larger sample was needed to demonstrate association between promoter CRLR gene variants with severe preeclampsia, anemia and other hypoxia condition. Further study is needed to demonstrate others DNA sequence motives in CRLR gene regulated CRLR receptor expression or other signaling that more dominant than adrenomedullin signaling in hypoxia condition.

## Supplementary Information

**Additional file 1: Figure S1.** CRLR Gene Sequencing. Red box showed sequencing analyzed of CACA box on our sample and blue box showed HRE sequences.

## Data Availability

Authors declare that the data will not be shared since they are patients’ confidentiality.

## Abbreviations

HRE: Hypoxia response element
HIF: Hypoxia induced factor (HIF)
CRLR: Calcitonin receptor-like receptor
STR: Short tandem repeat
HELLP: Hemolysis, elevated liver enzyme, and low platelet count syndrome
STR: Short tandem repeat
CGRP: Calcitonin gene related peptide
RAMP: Receptor activity modifying protein
EDTA: Ethylene diamine tetraacetic acid
PCR: Polymerase chain reaction
