# Supplementary figures and images for: Short tandem repeat near hypoxia response element (HRE) instead of HRE genetic variants in promoter calcitonin receptor-like receptor (CRLR) gene as risk factor in severe preeclampsia: a preliminary study
Source: BMC Res Notes. 2021 Jan 7;14:17. doi: 10.1186/s13104-020-05437-z (PMC7792221; doi:10.1186/s13104-020-05437-z)

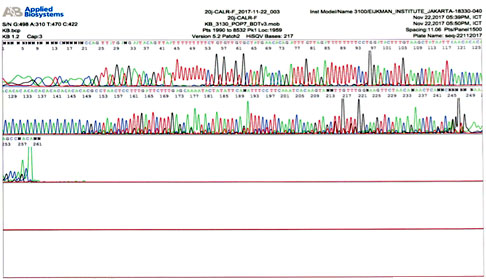

Supplement: Supplementary file 1 — Additional file 1: Figure S1. CRLR Gene Sequencing. Red box showed sequencing analyzed of CACA box on our sample and blue box showed HRE sequences. [file 13104_2020_5437_MOESM1_ESM.jpg]
